# Pulmonary exposure to carbonaceous nanomaterials and sperm quality

**DOI:** 10.1186/s12989-018-0242-8

**Published:** 2018-01-31

**Authors:** Astrid Skovmand, Anna Jacobsen Lauvås, Preben Christensen, Ulla Vogel, Karin Sørig Hougaard, Sandra Goericke-Pesch

**Affiliations:** 1 The National Research Center for the Working Environment, Lersø Parkallé, DK-2100 Copenhagen Ø, Denmark; 2 Section for Veterinary Reproduction and Obstetrics, Department of Veterinary Clinical Sciences, University of Copenhagen, Dyrlægvej 68, DK-1870 Frederiksberg C, Denmark; 3 SPZ Lab A/S, Fruebjergvej 3, DK-2100 København Ø, Denmark; 4 Department of Micro- and Nanotechnology, Technical University of Denmark, DK-2800 Kongens Lyngby, Denmark; 5 Department of Public Health, University of Copenhagen, Øster Farimagsgade 5, DK-1014 Copenhagen K, Denmark

**Keywords:** Nanomaterials, Particles, Toxicity, Semen parameters, Pulmonary exposure, Computer-assisted sperm analysis, Inflammation

## Abstract

**Background:**

Semen quality parameters are potentially affected by nanomaterials in several ways: Inhaled nanosized particles are potent inducers of pulmonary inflammation, leading to the release of inflammatory mediators. Small amounts of particles may translocate from the lungs into the lung capillaries, enter the systemic circulation and ultimately reach the testes. Both the inflammatory response and the particles may induce oxidative stress which can directly affect spermatogenesis. Furthermore, spermatogenesis may be indirectly affected by changes in the hormonal milieu as systemic inflammation is a potential modulator of endocrine function. The aim of this study was to investigate the effects of pulmonary exposure to carbonaceous nanomaterials on sperm quality parameters in an experimental mouse model.

**Methods:**

Effects on sperm quality after pulmonary inflammation induced by carbonaceous nanomaterials were investigated by intratracheally instilling sexually mature male NMRI mice with four different carbonaceous nanomaterials dispersed in nanopure water: graphene oxide (18 μg/mouse/i.t.), Flammruss 101, Printex 90 and SRM1650b (0.1 mg/mouse/i.t. each) weekly for seven consecutive weeks. Pulmonary inflammation was determined by differential cell count in bronchoalveolar lavage fluid. Epididymal sperm concentration and motility were measured by computer-assisted sperm analysis. Epididymal sperm viability and morphological abnormalities were assessed manually using Hoechst 33,342/PI flourescent and Spermac staining, respectively. Epididymal sperm were assessed with regard to sperm DNA integrity (damage). Daily sperm production was measured in the testis, and testosterone levels were measured in blood plasma by ELISA.

**Results:**

Neutrophil numbers in the bronchoalveolar fluid showed sustained inflammatory response in the nanoparticle-exposed groups one week after the last instillation. No significant changes in epididymal sperm parameters, daily sperm production or plasma testosterone levels were found.

**Conclusion:**

Despite the sustained pulmonary inflammatory response, an eight week exposure to graphene oxide, Flammruss 101, Printex 90 and the diesel particle SRM1650b in the present study did not appear to affect semen parameters, daily sperm production or testosterone concentration in male NMRI mice.

**Electronic supplementary material:**

The online version of this article (10.1186/s12989-018-0242-8) contains supplementary material, which is available to authorized users.

## Background

The use and development of nanotechnology have been rapidly increasing. The ever expanding application of nanomaterials (NMs) includes areas such as cosmetics, electronics and food science, and as a result, men in the reproductive age are potentially exposed to nanomaterials both as workers in the various industries and as consumers. Likewise, the general public can also be exposed due to the release of nanoparticles (NPs) into the environment from natural and anthropogenic sources. The male germ line is highly sensitive to toxic insults and a number of environmental toxicants, such as ionizing radiation, organic solvents, and heavy metals, markedly decrease semen quality [1]. The apparent worldwide decline in semen quality, a controversial and often debated statement, has been reported by several researchers [2–4]. Linear regression analysis of 138 published reports from Europe, North and South America, and Asia between 1980 and 2015 showed a 57% decrease in mean sperm concentration in men [4]. Danish researchers, for example, have reported a decreasing trend and although recent monitoring programs now document a slight increase in semen quality in young Danish men, only one out of four has optimal semen quality [3]. Air pollution and its particulate constituents have been associated with several adverse health effects, mainly pulmonary and cardiovascular diseases [5]. Epidemiological studies of adult men have, however,  also found that elevated levels of air pollution are associated with decreased sperm motility, increased percentages of morphologically abnormal sperm, and elevated levels of DNA damage in sperm [6, 7]. Consequently, the question of whether NMs can affect male fertility by decreasing semen quality parameters merits further investigation.

The mechanisms how inhaled NMs may affect semen quality are yet to be elucidated. It has been hypothesised that NMs may affect semen quality in several ways: when inhaled, particles are potent inducers of pulmonary inflammation, which may result in the release of inflammatory mediators into the blood stream. Small amounts of particles may also translocate from the lungs into the lung capillaries and enter the blood stream [8]. The systemic inflammation may weaken the integrity of the blood-testis-barrier and increase its permeability, ultimately allowing NMs that have deposited in the testis to enter the lumen of the seminiferous tubules [9]. An inflammatory response in the testis may be induced due to infection, trauma and/or environmental toxins. Accordingly, it may be possible that NMs in the testis may elicit a testicular inflammatory response and thus possibly activate resident macrophages or result in an influx of neutrophils and other leukocytes [10]. Particles and leukocytes may create a Reactive Oxygen Species (ROS)/antioxidant imbalance, as both the particles and the leukocytes are strong inducers of ROS [11]. High levels of oxidative stress have been hypothesised to be a major cause for male infertility, because spermatozoa are highly sensitive to oxidative damage [12].

Exposure to NMs may also indirectly affect spermatogenesis by affecting the hormonal milieu via effects on the hypathalamic-pituitay-gonadal axis, as this axis is sensitive to inflammation. In female mice, it has been recently shown that airway exposure to multi-walled carbon nanotubes can interfere with the estrous cycle by either direct action of the particles or indirectly by the influence of inflammatory and acute phase responses [13]. NP-rich diesel exhaust inhalation exposure (5 h/day, 5 days/week) of adult male Fisher 344 rats increased plasma testosterone levels, possibly due to the induction of testosterone biosynthesis through elevation of StAR and P450scc in the testis via growth hormone signaling. Interestingly, the NP-rich diesel exhaust did not show dose-dependent effects, high levels of testosterone were found at the low (2.27 × 10^5^ /cm^3^) and medium (5.11 × 10^5^ /cm^3^) exposure levels whereas testosterone concentrations remained unchanged at the high (1.36 × 10^6^ /cm^3^) exposure level [14]. In ICR mice, inhalation exposure (12 h/day for 6 months) to diesel exhaust at 0.3, 1 and 3 mg DEP/m^3^, has been shown to cause degenerative and necrotic changes in the testis, desquamation of the seminiferous tubules and loss of spermatozoa, degenerative changes in Leydig cells such as the appearance of myelin, lipid droplets and secondary lysosomes, and a reduction in daily sperm production (DSP) [15]. In the same strain, 10 weekly exposures to 0.1 mg/mouse by intratracheal instillation (i.t.) of three different sizes of carbon black (CB) NPs (14, 56, and 95 nm) were shown to significantly decrease DSP, increase testosterone levels and cause vacuolation of the seminiferous tubules [16]. Following intratracheal instillation of 2 mg/kg (every 3 days for 45 days) of silica particles (57 nm), particles have been observed to cross the blood-testis-barrier in C57BL/6 mice using transmission electron microscopy. The silica NPs decreased sperm concentration and motility, and increased sperm abnormalities. Testicular malondialdehyde and 3-nitrotyrosine levels were increased, whereas SOD activity was impaired; suggesting that the damage may have arisen due to oxidative stress in the testis [17].

Based on these findings, we hypothesised that airway exposure to nanomaterials may interfere with normal spermatogenesis and decrease the quality of sperm, potentially altering male reproductive function. To further investigate and characterize these effects, sexually mature NMRI male mice were exposed to four carbonaceus NMs with different shape, size and surface chemistry and the effects on sperm quality parameters and testosterone concentrations were investigated.

## Methods

### Experimental design

One hundred and five male NMRI mice, purchased from Taconic Biosciences Inc. (Ejby, Denmark), were acclimatized for one week prior to the commencement of the experimental procedures, which began when the mice were eight weeks of age. The mice were randomly divided into 7 groups (*n* = 15): graphene oxide, Flammruss 101, Printex 90, SRM1650b, vehicle (nanopure water) controls, unhandled controls and high fat diet (HFD) controls. The graphene oxide, Flammruss 101, Printex 90 and SRM1650b exposed animals were intratracheally instilled with 50 μl of particle suspension followed by 200 μl of air under general anesthesia with 3–4% isoflurane mixed with sterile filtered air as described by Jackson et al. [18]. The mice were instilled once a week for seven consecutive weeks and the study was terminated six to eight days after the last exposure resulting in a total exposure time of 1.6 spermatogenic cycles, as one spermatogenic cycle corresponds to ~35 days in mice. All the mice in the CB and diesel exhaust particle groups received the same dose of 0.1 mg/mouse per instillation, corresponding to a cumulative dose of 0.7 mg during the study period. The current occupational exposure limit in Denmark is 3.5 mg/m^3^ for CB. However, mean concentrations of 14.90 mg/m^3^ of CB have been measured by personal air samplers in the workplace [19]. Based on the observed particle size distribution during aerosolisation of particles [20], at the current occupational exposure limit of 3.5 mg/m^3^, the estimated deposited dose is 16.6 μg in mice, giving a weekly deposited dose of 83 μg. [20, 21]. The graphene oxide was administered at a lower dose of 18 μg/mouse per instillation, with a cumulative dose of 126 μg, to ensure that the animal’s welfare was not affected, based on previous findings [22]. The vehicle control group was treated as the particle exposed group and received instillations of 50 μl of nanopure water without NMs. The unhandled and HFD control groups did not receive instillations or isoflurane at any time. All 105 animals were randomly euthanized by exsanguination under deep anaesthesia with a cocktail of ZRF (Zoletil 250 mg, Rompun 20 mg/ml and Fentanyl 50 mg/ml in sterile isotone saline) at a dose of 0.01 ml per g body weight. Due to logistical reasons half of the mice in each group were euthanized six or eight days after the last instillation. Testicles and epididymides were collected and weighed separately. The right testicle was snap frozen in liquid nitrogen and the left testicle was stored in Bouin’s fixation solution. The head and tail of the epididymides were separated; the right and left head and the right tail were snap frozen individually. The left tail was used for sperm retrieval (see below).

The mice were housed individually in clear 1290D euro standard type 3 cages with aspen sawdust bedding (Tapvei, Estonia) and enrichment, nesting material (Enviro Dri, Lillico, Biotechnology, UK), mouse house (80-ACRE011, Techniplast, Italy) and small aspen blocks (Tapvei, Estonia). Housing conditions were kept constant, with a 12:12 h light and dark cycle at an average temperature of 22 °C and 55% humidity. Tap water and standard pellet diet Altromin no. 1324 (Brogaarden, Denmark) were provided ad libitum to all groups, except for the HFD control group which received a 60% kcal fat diet ad libitum upon arrival and throughout the study (RD Western Diet D12492, Open Source Diets, Brogaarden, Denmark). All experimental procedures followed the handling guidelines established by the Danish government and permits from the Experimental Animal Inspectorate (no. 201515–0201-00465 and 2015–15–0201-00569). Prior to the study, specific experimental protocols were approved by the local Animal Ethics Council.

### Nanoparticles, preparation and characterization

The physico-chemical properties of the studied particles have been assessed and reported previously [23–25] and are summarized in Table 1. The graphene oxide in aqueous suspension was manufactured and supplied by Graphenea (San Sebastian, Spain) and has been previously characterized in detail in Bengtson et al. [23]. In suspension it appears as flat plates consisting of mainly two to three stacked graphene layers with a lateral size of 2–3 μm. The specific surface area has not been reported, but the corresponding reduced graphene oxide had a specific surface area of 338–411 m^2^/g [23]. The Flammruss 101 and Printex 90 carbon black NPs in powder form were gifts from Boesens Fabrikker ApS (Denmark) and Degussa (Germany), respectively, and have been previously characterized in detail by Saber et al. [24]. Flammruss 101 consists of spherical particles with a primary particle diameter of 95 nm and a specific surface area of 23.8 m^2^/g [24]. Printex 90 has a similar shape to that of the Flammruss 101, with a reported primary particle diameter of 14 nm and a specific surface area of 295–338 m^2^/g [24]. The diesel exhaust particle (SRM1650b) is a standard reference material and the certificate of analysis is available from the National Institute of Standards & Technology (Gaithersburg, MD, USA, https://www.nist.gov/). It is an exhaust particle from a heavy duty diesel engine with a reported primary particle diameter of 18–30 nm and a specific surface area of 108 m^2^/g [25]. Unlike the other three particles, the SRM1650b has a high content of adhered heavy metals and polycyclic aromatic hydrocarbons (PAHs) i.e. a ~3000 fold higher content of PAHs compared to Printex 90 [25]**.**

**Table 1:** Summary of particle characteristics

Particle	Primary particle size	Shape	Surface area	Z-average (in nanopure water)	Reference
Graphene oxide	Lateral size: 2–3 μm Thickness: 2 nm	flat plates consisting of mainly two to three stacked graphene layers	338–411 m^2^/g (reduced graphene oxide)	486.7 nm	[31]
Flammruss 101	95 nm	spherical	23.8 m^2^/g	305.4 nm	[32]
Printex 90	14 nm	spherical	295–338 m^2^/g	147.2 nm	[32]
SRM1650b	18–30 nm	spherical	108 m^2^/g		[33]

For instillation, the particles were dispersed in nanopure water at a concentration of 2 mg/ml and sonicated for 16 min on ice using a 400 W Branson Sonifier A-450D (Branson Ultrasonic Corp., Danbury, CT, USA) equipped with a disruptor horn (Model 101–147-037). The hydrodynamic particle size distribution in nanopure water was measured by dynamic light scattering using a Malvern Zetasizer Nano ZS equipped with a 633 nm He-Ne Laser (Malvern Inc., UK).

### Bronchoalveolar lavage

Bronchoalveolar lavage fluid (BALF) differential cell counts were performed as previously described in Kyjovska et al. [26]. The BALF was collected for 12 of the 15 mice per particle exposed group as the lungs of 3 mice per group were collected for histology. The trachea of the mice (*n* = 12 per group) was exposed and cannulated with a 22 gauge BD Insyte catheter. The lungs were flushed twice with 0.8 ml of 0.9% saline in a 1 ml syringe. The BALF was centrifuged at 400 g at 4 °C for 10 min. The cell pellet was re-suspended in 100 μl of Ham’s F-12 Nutrient Mix cell culture medium. Total cells were counted using a NucleoCounter (Chemometec, NucleoCounter NC-200). For the differential cell counts 50 μl of the BALF cell suspension were pipetted onto glass slides and spun at 1000 rpm for 4 min in a cytospin centrifuge. The slides were fixated and then stained with May-Grünwald Eosin-Methyleneblue and Giemsa Azur-Eosin-Methylene Blue solution. Differential cell counts were performed under a bright field microscope using oil immersion and a 1000× magnification.

### Collection of epididymal sperm and computer-assisted sperm analysis of concentration, motility and viability

The left epididymal tail was placed in 500 μl warm (37 °C) TCM199 medium (Sigma-Aldrich, Denmark) and minced with scissors. The sperm cells were allowed to swim out for 10 min and were then filtered through a stainless steel mesh. Samples were kept at 37 °C on a heating stage during the whole procedure including microscopy analysis. Computer-assisted sperm analysis (CASA) was performed using a negative phase contrast microscope (Olympus BX60, Tokyo, Japan) equipped with a heating stage and a high-speed GigE camera (avA21000-100gc) with a CCD sensor (aviator series, Basler, Germany) detecting 101 frames/s and the AndroVision software (Ref 12,500/0000, Software Version 1.0.0.9, Minitube, Tiefenbach, Germany). For analysis of concentration and motility, an aliquot of the diluted semen (2.0 μl) was pipetted into an evaluation chamber (Leja ® Standard Count 4 Chamber Slide, 10μm, Leja Products B. V., Nieuw Vennep, The Netherlands) and 10 randomly distributed fields were analysed at 200× magnification. The software calculated the sperm concentration per mL and analysed the sperm motility parameters. Motility results were presented as the total percentage of motile spermatozoa and the percentage of progressively motile spermatozoa. The following settings on the CASA system were used: sperm recognition area 10–100 μm^2^, 10 fields per sample, TM = PM + LM, PM = CM+ slow motility + fast motility, LM: velocity curved line (VCL) < 80 × 10^4^μ/s and velocity straight line (VSL) < 20 × 10^4^μ/s, Circular Motility: linearity <0.6000 and rotation >0.8000.

Additionally, another aliquot (50 μl) of the diluted semen was added to 1.5 μl of ready to use Hoechst 33,342/PI fluorescent stain (Minitube) and incubated at 37 °C for 15 min. The viability was analyzed manually by counting 200 sperm per sample using a fluorescent microscope fitted with a U-MU filter cube and a mercury burner. The results were presented as percentage of viable spermatozoa. Blue sperm were considered as viable, whereas red were considered as non-viable (www.minitube.com).

### Sperm morphology

Native semen smears were prepared, air-dried, fixated and stained with Spermac® according to the manufacturer’s instructions (Minitube). 200 spermatozoa were identified and categorized as normal or as having a morphological deviation. Deviations were differentiated into sperm acrosome, head, neck, mid-piece or tail defects, cytoplasmic droplets or loose heads. In case of several morphological deviations in one sperm, only the one considered as the most severe was recorded. The exposure status of the samples on morphology was blinded to the scorer. The results were presented as percentage of abnormal spermatozoa in the respective location as well as total percentage of normal spermatozoa as defined by 100% - each % of abnormalities in the respective locations.

### Sperm DNA integrity

Neat epididymal semen samples were diluted 1:2 with TNE buffer (0.01 M Tris-Cl, 0.15 M NaCl, 1 mM EDTA, pH 7.4) and frozen directly at −196 °C in a dryshipper and transported to the laboratory. The fluorescent staining was performed according to the protocol for the sperm chromatin structure assay as described by Evenson and Jost 2000 [27]. Semen samples were thawed at 35 °C for 3 min and were then incubated on ice for 5 min [28]. An aliquot of the thawed sample was diluted to a concentration of 2 × 10^6^ sperm/mL with TNE buffer to a total volume of 200 μL. DNA denaturation was induced by addition of 400 μL acid detergent solution (0.08 M HCl, 0.15 M NaCl, 0.1% *v*/v Triton X-100, pH 1.2). After 30 s, 1.20 mL of acridine orange staining solution (Citric acid 0.037 M, Na_2_HPO_4_ 0.126 M, NaCl 0.15 M, Na_2_EDTA 1 mM, pH 6.0) were added. The sample was immediately placed in the flow cytometer and run through the system to allow for equilibration prior to acquisition of data. The samples were blinded and analyzed using a FACSCalibur (BD Biosciences) flow cytometer with an air-cooled argon orthogonal laser operating at 488 nm with 15 mW of power. After transiting a 560 nm short-pass dichroic mirror, the green fluorescence (FL1) was collected through a 515 to 545 nm band-pass filter and the red fluorescence (FL3) through a 650 nm long-pass filter. The sheath/sample was set to “high” with an estimated flow rate of 60 μl/min. This flow rate resulted in analysis of approximately 200 events per second. Acquisition of 5000 events was started exactly 3 min after initiation of the acid detergent treatment at a point in time when the sample had been running through the flow system for approximately 2.5 min to achieve equilibration. To ensure good quality control, each analysis was run in duplicate and results were only accepted, if the standard deviation (SD) between duplicates was below 2.5%. If variation exceeded 2.5%, two new aliquots were analyzed. If the event rate was above the expected 200 events per second, a new dilution and staining cycle was performed to ensure an event rate below 200 and thus an optimal ratio between acridine orange molecules and DNA. The results of the analyses were reported as DFI% which describes the proportion of sperm with a detectable level of DNA damage after acid denaturation.

### Daily sperm production

The adipose tissue from the frozen testes was trimmed and the tunica albuginea was peeled off with forceps after making a shallow longitudinal incision. The testes were weighed, placed into 4 ml of 0.05% TRITON-X100 and homogenized for 3 min using the IKAULTRA TURRAX T25 disperser S25 N-10G. Homogenates were kept on ice for 30 min. 200 μl of the homogenate were mixed with 200 μl of 0.04% Trypan blue and left for 5 min at room temperature. Sperm heads were counted using a Bürker counting chamber. DSP was calculated using the following formulas:

N = sperm number per μl x volume of lysis (buffer)

DSP = N / 4.84

where N is the total number of spermatids per sample. The DSP is then calculated by dividing the total number of spermatids per sample by 4.84, which is the number of days for a spermatid to develop through stages 14 to 16, i.e., the stages where spermatids are resistant to homogenization. The samples were blinded and counts were done in duplicates. If the two counts deviated by more than 20%, the procedure was repeated for the sample.

### Testosterone measurement

Blood was collected from the heart, stabilized using K_2_EDTA and then centrifuged at 2500 g for 10 min. The EDTA-plasma was pipetted into separate snapstrip PCR-vials and stored at −80 °C until analysis. The plasma samples were blinded and the testosterone concentrations were determined in duplicates and 1:2 dilutions with phosphate-buffered saline (PBS), using competitive ELISA (RTC001R, Biovendor, Brno, Czech Republic). Samples were analysed following the manufacturer’s protocol, with a standard curve in the range of 0.1–25 ng/mL. All samples that fell outside the standard curve were diluted 1:4 in PBS and re-analyzed. (Interassay) coefficient of variance was 4.8–7.8%.

### Statistical analysis

An ANOVA was used to test for the overall significance of the BALF counts and was followed by a Dunnett’s test where particle exposed and unhandled control groups were compared to the vehicle control (SAS® software, version 9.4 of the SAS system for windows 7 (Cary, NC, USA)). All other data were analyzed by ANOVA, followed by post-hoc Fischer least statistical difference test when appropriate (Origin Pro, version 2016 (64-bit), OriginLab Corp (Northampton, MA, USA)). Results obtained from mice exposed to NMs were compared to those from vehicle-exposed mice, whereas results from HFD mice were compared to those of unhandled controls. The level of significance was set at 0.05. The *a priori* statistical power analysis had been calculated using the 33% ± SD difference in DSP between the Printex 90 and vehicle control exposed mice reported by Yoshida et al. 2008 (16) (G*Power software version 3.1.9.2, Düsseldorf, Germany).

## Results

### Nanoparticle characterization

The graphene oxide, Flammruss 101 and Printex 90 dispersed in nanopure water at a concentration of 2 mg/ml had a Z-average of 486.7 nm, 305.4 nm and 147.2 nm, respectively. Due to a lack of material the DLS was not performed on the SRM1650b, however, the SRM1650b dispersed in nanopure water at a concentration of 3.24 mg/ml was previously measured to have a Z-average of 167.8 nm (25). The particles characteristics are summarized in Table 1.

### Body, testicular and epididymal weights

There was no difference in body weight and absolute and relative organ weight between the groups, except for the HFD controls which had a statistically significantly higher body weight compared to all other groups (Table 2). However, only five out of the 15 mice in the HFD group gained enough weight to be considered obese, which means mice weighing more than 51.27 g, based on the mean weight of the unhandled +2 SD.

**Table 2:** Body weight (g), and absolute (mg) and relative weights of left testis and epididymis at the time of euthanasia

	Body weight (g)	Testis weight (mg)	Testis relative weight	Epididymal weight (mg)	Epididymal relative weight
Vehicle control	39.39 ± 3.19	107.67 ± 6.61	2.75 ± 0.23	45.33 ± 6.21	1.15 ± 0.14
Graphene oxide	40.26 ± 3.82	103.10 ± 14.03	2.58 ± 0.37	43.29 ± 6.33	1.08 ± 0.14
Flammruss 101	39.91 ± 4.23	107.91 ± 15.43	2.71 ± 0.42	39.49 ± 9.32	0.98 ± 0.18
Printex 90	37.96 ± 2.15	107.91 ± 10.34	2.85 ± 0.25	43.71 ± 3.97	1.15 ± 0.11
SRM1650b	40.08 ± 3.50	103.14 ± 17.46	2.58 ± 0.42	41.09 ± 12.41	1.09 ± 0.13
Unhandled	42.97 ± 4.15	107.78 ± 11.14	2.53 ± 0.32	45.03 ± 2.48	1.06 ± 0.13
High fat diet	50.09 ± 6.34^a^	111.77 ± 7.45	2.26 ± 0.28	46.16 ± 3.09	0.93 ± 0.12

Mean ± SD (*n* = 15).

^a^ < 0.001, compared with vehicle control

### Pulmonary inflammation

BALF neutrophil numbers were significantly elevated in the lungs from mice exposed to graphene oxide (51-fold increase), Flammruss 101 (61-fold increase), Printex 90 (329-fold increase) and SRM1650b (78-fold increase) compared to vehicle controls (*p* < 0.001) (Table 3). The unhandled and the HFD were not statistically different from the vehicle control group, confirming that the instillation procedure and the vehicle did not induce pulmonary inflammation. Interestingly, Printex 90 induced a stronger inflammatory response one week after the last instillation compared to the other three NMs. Neutrophil influx was plotted against deposited surface area since surface area may be a more biological relevant dose metric for spherical NMs than mass (see Additional file 1). Neutrophil cell numbers correlated with deposited surface area (R^2^ = 0.64).

**Table 3:** Pulmonary inflammation presented as total cell, macrophage and neutrophil counts in the BALF 6 to 8 days after the last instillation

	Total cells (×10^5^)	Macrophages (×10^5^)	Neutrophils (×10^5^)
Vehicle control	10.95 ± 0.73	10.58 ± 0.73	0.04 ± 0.02
Graphene oxide	16.21 ± 1.15^a^	13.39 ± 0.97	2.07 ± 0.39^b^
Flammruss 101	11.89 ± 2.09	8.85 ± 0.79	2.47 ± 1.46^b^
Printex 90	27.90 ± 2.96^b^	19.09 ± 2.06	13.19 ± 1.11^b^
SRM1650b	14.78 ± 1.24	10.90 ± 0.83	3.15 ± 0.56^b^
Unhandled	12.56 ± 1.25	12.07 ± 1.24	0.11 ± 0.02
High fat diet	10.92 ± 0.91	10.18 ± 0.92	0.07 ± 0.03

Mean ± SD (*n* = 13–15)

^a^ < 0.05, ^b^ < 0.001, compared with vehicle control

### Epididymal sperm concentration, motility, viability, morphology and sperm DNA damage

**Fig. 1:**
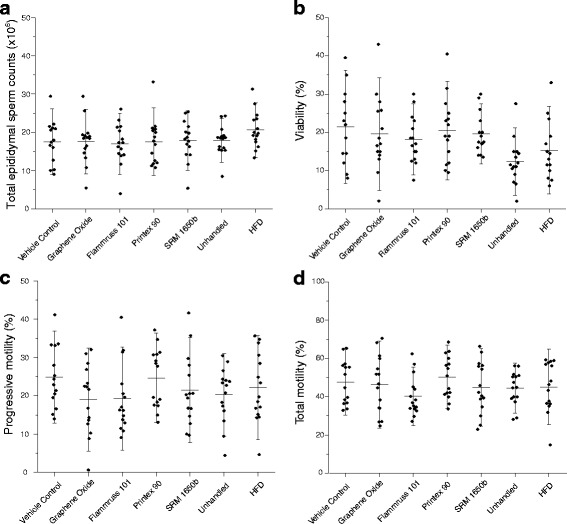
Epididymal sperm parameters analysed from the left epididymal tail. **a** Total epididymal sperm counts (×10^6^) **b** Viable sperm (%) **c** Progressive motility (%) **d** Total motility (%). Mean ± SD (*n* = 14–15)

**Fig. 2:**
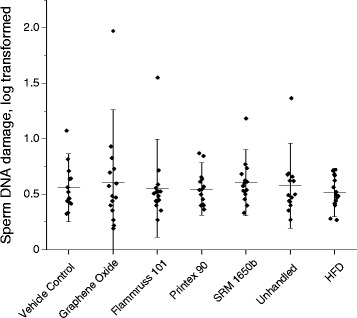
DFI (Sperm DNA damage, log transformed). Mean ± SD (*n* = 15)

There was no statistically significant difference between the groups for the epididymal sperm concentration, total and progressive motility, viability (Fig. 1) and sperm DNA damage (DFI, Fig. 2). Furthermore, there was no significant difference between the groups regarding sperm morphology (percentage of sperm acrosome, head, neck, mid-piece and tail defects, cytoplasmic droplets and loose heads) (Table 4).

**Table 4:** Percentages of normal spermatozoa and of spermatozoa with morphological defects in the acrosome, head, neck, mid-piece or tail region, those having a cytoplasmic droplet and a loose head

	Normal (%)	Acrosome (%)	Head (%)	Neck (%)	Mid-piece (%)	Tail (%)	Cytoplasmic droplets (%)	Loose heads (%)
Vehicle control	30.64 ± 10.0	16.7 ± 10.3	3.00 ± 2.5	5.39 ± 2.4	3.07 ± 3.8	33.54 ± 8.2	3.29 ± 4.8	4.32 ± 3.5
Graphene oxide	24.50 ± 13.5	12.17 ± 12.2	2.77 ± 2.7	7.77 ± 7.7	4.83 ± 7.1	39.57 ± 15.2	2.37 ± 2.3	6.03 ± 5.6
Flammruss 101	29.86 ± 8.1	17.32 ± 12.1	4.32 ± 2.5	4.68 ± 2.4	0.82 ± 1.0	35.36 ± 12.0	2.28 ± 3.1	5.35 ± 2.5
Printex 90	28.23 ± 10.7	21.10 ± 9.6	2.56 ± 1.2	5.07 ± 3.3	2.00 ± 1.9	32.20 ± 12.4	2.17 ± 3.0	6.67 ± 3.9
SRM1650b	26.26 ± 9.5	23.50 ± 7.9	4.76 ± 4.1	6.17 ± 3.0	2.30 ± 1.7	29.23 ± 11.7	1.43 ± 1.7	6.33 ± 5.4
Unhandled	25.20 ± 12.8	18.87 ± 9.7	1.70 ± 1.2	4.37 ± 2.0	0.83 ± 1.0	36.83 ± 10.5	1.00 ± 1.5	10.80 ± 16.9
High fat diet	26.63 ± 10.1	21.56 ± 10.4	3.63 ± 1.6	4.46 ± 2.3	1.47 ± 1.5	36.16 ± 9.12	1.33 ± 1.4	4.73 ± 2.8

Mean ± SD (*n* = 14–15)

### Daily sperm production and testosterone

**Fig. 3:**
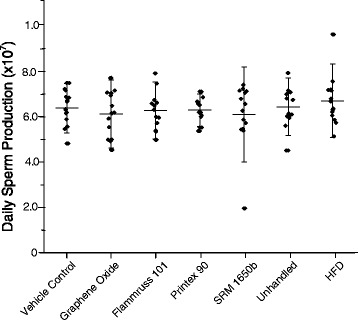
Daily sperm production derived as spermatids in developmental stage 14 to 16 measured in the left testicle (×10^7^ spermatids). Mean ± SD (*n* = 13–15)

**Fig. 4:**
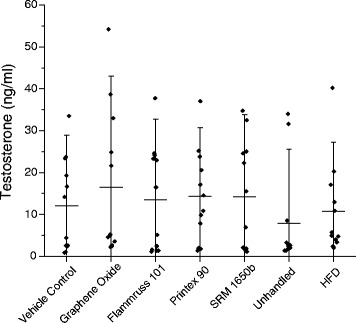
Testosterone concentration (ng/ml) in plasma. Mean ± SD (*n* = 13–15)

There was no statistically significant difference between groups for DSP (Fig. 3) and blood plasma testosterone concentrations (Fig. 4).

## Discussion

Knowledge about the effect of NMs on sperm quality is limited, especially following pulmonary exposure. Although various NMs have been reported to induce testicular toxicity and decrease semen quality, mainly oral and intravenous exposures have been investigated [9, 29]. This is somehow striking as inhalation is the major exposure route for NMs in humans, and the systemic inflammation which is induced after a pulmonary exposure may be an important indirect mechanism for the induction of testicular toxicity. In the current study, male mice were exposed via the lungs to four different carbonaceous NMs and the effects of these NMs on sperm quality parameters, measured as epidydimal sperm concentration, viability, motility, morphology, sperm DNA damage, DSP, and plasma testosterone concentrations, were investigated.

At a final cumulative dose of 700 μg/mouse (126 μg for graphene oxide), the significant influxes of PMNs demonstrate pulmonary inflammation one week post exposure compared to the three control groups. Previous studies showed that instilled Printex 90 at a final cumulative dose of 268 μg/mouse induced lung inflammation in terms of increased neutrophil influx and expression of inflammatory and acute phase response both at mRNA and protein levels in the lung, including increased expression of chemokine ligand 5 (Cxcl5), serum amyloid A 3 (Saa3), immunoglobulin joining chain (Igj) and lymphocyte antigen 6 complex, locus F (Ly6f) [30]. This reflects that at high levels of CB exposure there is a pro-inflammatory response and an adaptive immune response. Based on our previous results, the observed neutrophil influx would suggest systemic inflammation at the applied cumulative dose levels. Despite the pulmonary inflammatory response we did not identify statistically significant differences in the investigated sperm parameters and testosterone concentrations between the particle exposed and vehicle control group.

Our results are in direct contrast to those described by Yoshida et al. (2008) who, at similar dose levels as in the present study, reported reduced DSP, seminiferous tubules damage and increased testosterone concentrations in ICR mice following ten instillations of Printex 90, and reduced DSP and seminiferous tubules damage following ten instillations of Flammruss 101 NPs [16]. The reason for this discrepancy is not clear. There are, however, major differences between the two studies in reference to mouse strain (NMRI versus ICR), number of instillations (seven versus ten), sampling time (24 h versus 6–8 days after the last instillation), and the vehicle used for the dispersion of particles (nanopure water versus saline with 0.05% tween 80).

The use of different vehicles and dispersants warrants important consideration in studies of male reproductive toxicity of NMs, because the vehicle can potentially change the chemical and physical properties of the particles and thereby influence their bioavailability and thus their potential for toxic insult. Surfactant molecules, like tween, have both lipophilic and hydrophilic properties and are therefore able to partition between lipid and protein structures; they are also known to enhance permeability because of their effects on tight junctions and cellular membranes [31]. Studies in male mice dosed intravenously with graphene oxide (1000 μg/ml) with or without 1% tween 80 showed that graphene oxide alone appeared to have a higher retention in the lungs compared to the graphene oxide with tween 80. In contrast, increased amounts of graphene oxide with tween 80 appeared to be retained in the liver. The authors concluded that tween 80 changes the zeta potential of particles and enables particles, like graphene oxide, to pass the capillary bed without massive deposition in the lungs [32]. Interestingly, no aggregates of graphene oxide or histopathological changes were found in the testis from mice in any of the graphene groups [32]. Nevertheless, Akhavan et al. [33] found accumulation of graphene oxide in the testis accompanied by significantly decreased epididymal sperm viability and motility, and increased sperm DNA damage and ROS generation in semen after an intravenous administration to BALB/c mice at a dose of 4 mg/kg of graphene oxide dispersed in PBS and DSPE-PEG-NH_2_ polymers. On the other hand, Liang et al. [34] found that intravenous administration of graphene oxide dispersed in PBS alone at 6.25, 12.5 and 25 mg/kg to ICR mice had no effect on epididymal sperm motility, morphology, concentration, male endogenous sex hormone and histology in the testis. Similarly, 10 instillations of Printex 90 dispered in 0.05% tween 80 caused adverse effects on reproductive parameters [16], whereas 7 instillations of Printex 90 dispered in nanopure water alone did not (present study). Translocation of titanium dioxide nanoparticles from lung to secondary tissues including liver and heart has been shown after intratracheal instillation of nano-TiO_2_ dispersed in 0.9% NaCl MilliQ water with 10% acellular BAL fluid [35] or water [36]. Therefore, we might expect some degree of translocation into the systemic circulation of nanosized particles that were deposited in the lungs. However, in the present study, microscopical examination of the testis revealed no gross morphological alterations between the groups and there was no apparent indication of particle deposition in the testis (data not shown). A more comprehensive comparison on the potentially increased bioavailability to reproductive organs and the potential disruption of the blood-testis-barrier as well as testicular toxicity of surfactant coated and non coated NMs remains to be investigated.

Apart from surface coating, other physicochemical properties of the nanomaterials, such as size and core chemistry, may influence their effects on the male reproductive system [37]. Size-dependent effects on plasma testosterone are apparent in the paper by Yoshida et al. (2008), as plasma testosterone was increased for Printex 90 (primary particle size of 14 nm) while it remained unchanged for Flammruss 101 (primary particles size of 95 nm). We failed to reproduce this dependency on particle size, despite the evident differences in primary particle size of the carbonaceous NMs as well as the particle-induced inflammation.

In the present study, the nanomaterials were deposited in the lungs by instillation, i.e., the materials were delivered as a bolus. This typically results in a higher dose rate than during inhalation, and instillation may therefore not compare directly to real-life exposure. Instillation is very convenient for conduction of proof of principle studies and comparison of toxicity between studies and particles, as it ensures that similar doses can be delivered for all assessed particles. For Printex 90, we have, however, previously shown that inhalation and instillation may both induce strong and long lasting lung inflammation at estimated comparable deposited dose levels [20]. Furthermore, studies of the pulmonary global transcriptional responses following inhalation and pulmonary exposure to two different nanomaterials suggest that the global transcriptional responses to inhaled and instilled or aspirated nanomaterials are very similar [38, 39].

Spermatogenesis is a steady state process and the ability to regenerate germ cell populations and recover functional spermatogonia after toxic insult are good. In fact, full recovery after an intratracheal instillation of 2 mg/kg of micelle coated silica NPs (57.66 nm) dispersed in saline has been observed [17]. Approximately thirty days after the last exposure, TEM images revealed that the silica particles could no longer be observed in the testis of C57 mice, and the reduced sperm motility and increased sperm abnormalities and apoptosis had been reversed [17]. Potentially, induced effects may have been reversed in our study one week after the last instillation, when the tissue samples were collected. However, at the time of necropsy, the observed pulmonary inflammation indicated pulmonary presence of the particles. Pulmonary translocation of NMs is an ongoing process and would still be occuring for days following the last instillation. Time dependent translocation has been shown in rats. Hence instilled radioisotopes of nanosized Cerium-141 were measured at significantly higher levels 28 days post-instillation in the blood, liver and spleen compared to day seven post-instillation [40]. With regards to time-dependent translocation to the testis, multiwalled carbon nanotubes dispersed in PBS and 0.1% tween 80 administered intravenously at a dose of 5 mg/kg to BALB/c mice showed an increased trend of translocation to the testis; 41, 61 and 151 ng were found in the testis 10 min, 60 min and 24 h post-exposure, respectively. The authors concluded that after repeated administration the multiwalled carbon nanotubes would continue to accumulate in the testis, and certain effects could be observed up to 15 days post-instillation. Furthermore, during week eight (day 56) when our experiment was terminated and organs were collected, the spermatids in the epididymides would correspond to those in the testis during the first and second instillations, as spermatogenesis in mice takes 35 days plus approximately 14 days for epididymal maturation. We therefore postulate that if there would have been significant testicular toxicity, either by direct effect of the particles or indirectly by the inflammation, it would have been detected one week after the last instillation in the present study.

The HFD was chosen as a positive control because it has been previously shown to have a negative impact on semen quality in mice, e.g. by decreasing sperm motility, increasing oxidative stress (measured by intracellular ROS) and increasing sperm DNA damage [41]. However, the selection of a HFD as a positive control is a critical limitation to the study. Only one third of the mice in the HFD control group gained sufficient weight to be regarded as obese and therefore the effects, for examples on motility, may have not been detected (see Additional file 2). Suceptibility to HFD based adipose tissue inflammation and lipid peroxidative damage in muscle and liver have been shown to be strain specific [41]. In addition, it has been previously reported that semen quality and suceptibility to toxic insult may vary greatly between mouse strains [42, 43]. For example, the inflammatory marker TNF-α was significantly upregulated in the epididymal adipose tissue of BALB/c and FVB/N mice fed a HFD, while TNF-α remained unchanged in BL/6, 129/X1 and DBA/2 mice fed with the same diet [41]. The use of different mouse strains and experimental models in studies of male reprotoductive toxicity may provide some explanation to the contradictive results often encountered in this field.

To our knowledge, the present study is the first to investigate male reproductive toxicity of carbonaceous NMs administered via the lung, without the use of surfactants like tween 80. The strength of the study is that all instilled mice, including the vehicle controls, underwent the same exposure procedure and received the same vehicle. We are therefore confident that there is no added effect from the procedure or the choice of vehicle, as confirmed by the similar low levels of neutrophils in the vehicle control compared to the unhandled and HFD groups reciving no instillation. Several of the assays presented here, such as the DSP [44], testosterone ELISA (unpublished data), and DNA damage [27, 28] assays were validated prior to this experiment. *An a priori* power analysis indicated that the chosen group size in this study (*n* = 15) provided a 95% chance of detecting approximately a one-fold difference at the 5% significance level.

## Conclusion

In the present experiment, our results suggest that sperm quality parameters (epidydimal sperm concentration, sperm viability, sperm motility, sperm morphology, sperm DNA damage, DSP, and plasma testosterone concentration) were not altered in the exposed groups compared to the controls, neither by direct action of the NMs nor indirectly from the inflammatory response, after eight weeks of exposure to graphene oxide (18 mg/mouse/i.t.), Flammruss 101, Printex 90 and the SRM1650b (each 0.1 mg/mouse/i.t.) dispersed in nanopure water, in the NMRI mouse model. Standardization of experimental procedures, e.g. use of vehicle, in studies of male reproductive toxicity of NMs are needed in order to have a collective conclusion on the effects of NMs on male reproductive function. This may be imperative when determining legislative measures on workplace exposure levels for men in the reproductive age.

## Additional files

Additional file 1: Deposited surface area PMN. Neutrophil influx plotted against deposited surface area. (PDF 5 kb)

Additional file 2: Weight vs motility. Plot showing inverse correlation between progressive motility and body weight of mice. (PDF 7 kb)

## Abbreviations

BALF: Bronchoalveolar lavage fluid
CASA: Computer assisted sperm analysis
CB: Carbon black
DSP: Daily sperm production
HFD: High fat diet
i.t.: Intratracheal instillation
NMs: Nanomaterials
NPs: Nanoparticles
PAH: Polycyclic aromatic hydrocarbons
PBS: Phosphate-buffered saline
ROS: Reactive oxygen species
SD: Standard deviation
SRM: Standard reference material
