# Supplementary figures and images for: Pulmonary exposure to carbonaceous nanomaterials and sperm quality
Source: Part Fibre Toxicol. 2018 Jan 31;15:10. doi: 10.1186/s12989-018-0242-8 (PMC5793436; doi:10.1186/s12989-018-0242-8)

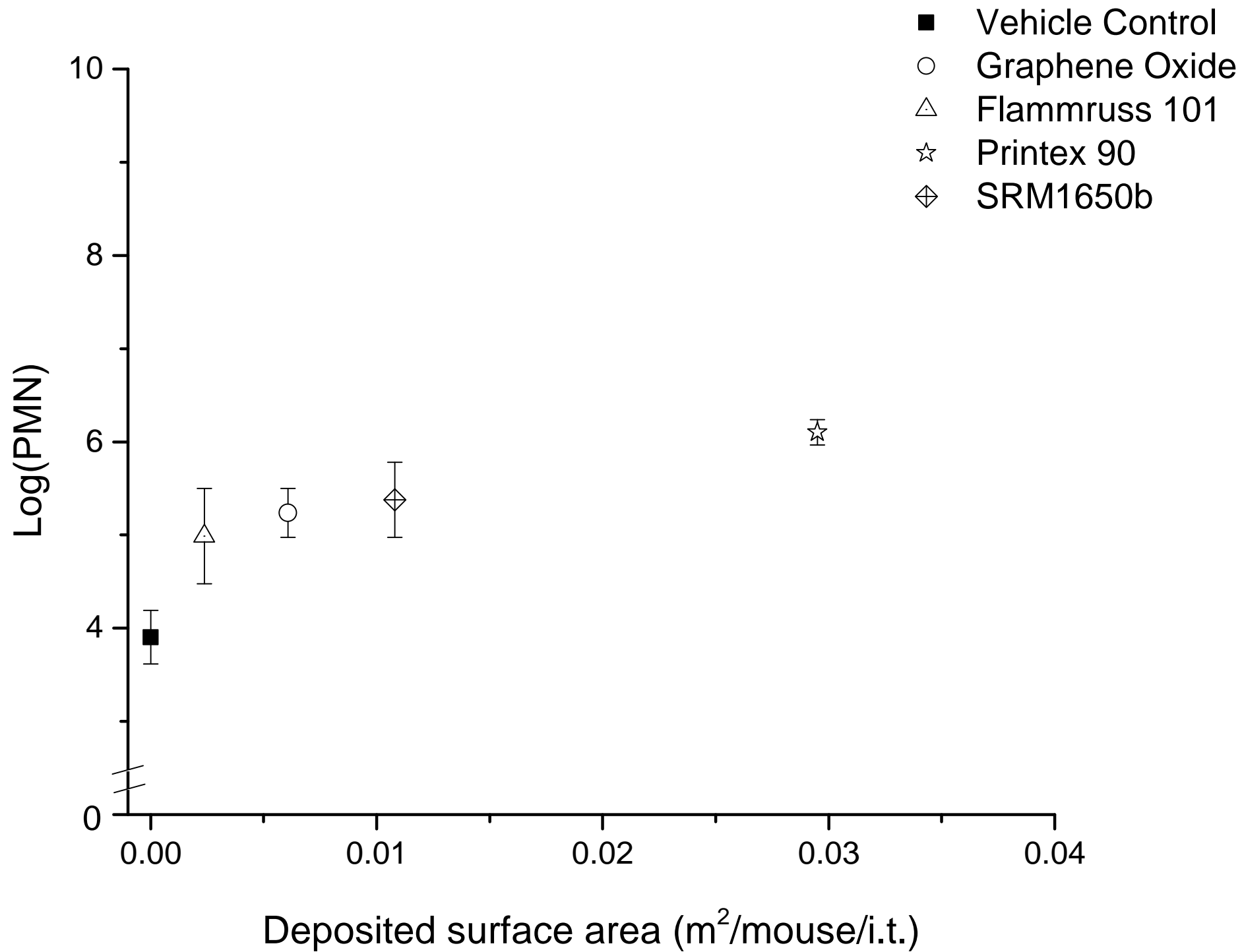

Supplement: Supplementary file 1 — Deposited surface area PMN. Neutrophil influx plotted against deposited surface area. (PDF 5 kb) [file 12989_2018_242_MOESM1_ESM.pdf]

Intercept = 45.69775, Slope = -0.48527  
X Intercept = 94.17022

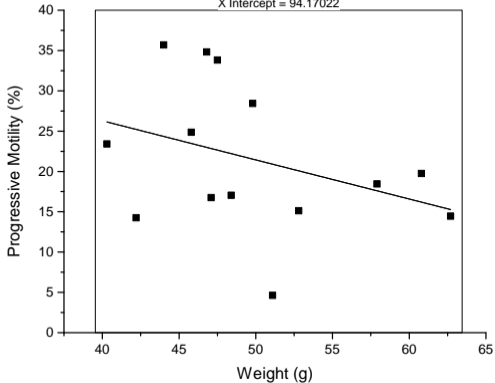

Supplement: Supplementary file 2 — Weight vs motility. Plot showing inverse correlation between progressive motility and body weight of mice. (PDF 7 kb) [file 12989_2018_242_MOESM2_ESM.pdf]
